# Preliminary observations on the plasma composition of *liza klunzingeri* from the strait of Hormuz (Persian gulf)

**DOI:** 10.1186/2193-1801-2-62

**Published:** 2013-02-21

**Authors:** Maria Mohammadizadeh, Majid Afkhami, Kazem Darvish Bastami, Maryam Ehsanpour, Reza Esmailpoor

**Affiliations:** Department of environmental management, Islamic Azad University, Bandar abbas branch, P.O.Box:79159–1311, Bandar abbas, Iran; Islamic Azad University, Bandar Abbas Branch, P.O.Box:79159–1311, Bandar Abbas, Iran; Iranian National Institute for Oceanography (INIO), P.O. Box 46515–369, Tehran, Iran

**Keywords:** *Liza klunzingeri*, T3, TSH, AST, ALT, Hormoz strait

## Abstract

Serum biochemistry can be used for monitoring changes in the physiological condition of fish and water quality. The aim of this paper was to determine the concentrations of plasma T3 (Thyroid hormone), thyroxin, TSH (Thyroid-stimulating hormone), ALT (Alanine Aminotransferase), AST (Aspartate aminotransferase) and cholesterol of *Liza klunzingeri* caught on the northern side of the strait of Hormuz (Persian Gulf). Biochemical values were: T3 0.96 ± 0.58 ng/ml, Thyroxin 76.58 ± 28.26 ng/ml TSH 0.03 ± 0.01 nmol/L, ALT 1.71 ± 0.68 U/L, AST 49.81 ± 5.25 U/L and cholesterol 177.28 ±40.75 mg/di. A significant positive correlation (P < 0.01) was found between AST and Cholesterol. ALT had a significant and positive correlation with cholesterol and AST (P < 0.01). Thyroxin also had a significant and positive correlation with cholesterol (P < 0.01) and AST (P < 0.01). The results revealed negative correlation between Thyroxin with TSH (P < 0.01). This study provides the first data on this blood chemistry of *L. klunzingeri*.

## Introduction

Blood analysis is a useful tool for the diagnosis and health monitoring of animals, as well as to distinguish pathogenic processes from those that might be purely physiological (Christopher et al., [1999]). Fish culturists and fish biologists use indices of blood chemistry for evaluating of fish stress responses, nutritional condition, reproductive state, tissue damage due to handling procedures, and health status (Wagner and Congleton, [2004]). It is well-known that blood sampling, laboratory techniques, seasonal variations, fish size, genetics patterns, stocking density, food privation, social stress, capture, handling and transport can all influence the biochemical parameters (Rey Vazquez and Guerrero, [2007]).

The Persian Gulf is a rich area for fishery resources, and large quantities of fish and shrimps are concentrated in different locations, particularly along the northern side of the Strait of Hormuz. *Liza klunzingeri* is a shared stock that is native to the Arabian Sea, Indian Ocean, Gulf of Oman and Persian Gulf (Randall, [1995]). The abundance of grey mullets in estuarine and coastal areas of tropical, sub-tropical and temperate regions of the world may be related to their food and feeding habits, as they occupy a relatively low position in the food web (Wright, [1988]). Despite their worldwide commercial importance, only very limited and disparate information exists on any aspect of the biochemical values in the blood of *Liza klunzingeri* either locally or regionally (Mohammadizadeh et al., [2012]). Normal reference ranges of blood biochemical parameters are considered important for assessing and monitoring the health status of *Liza klunzingeri*.

## Material and methods

*L.* (*klunzingeri* wild samples (n = 87) were captured with a kind of coastal trap net fishing gear (Mushta in local language) from northern side of Strait of Hormuz in 2011. Each fish was quickly caught and while it was physically restrained, a blood sample was collected directly from the heart with syringes coated with sodium heparin. These samples were used for determining the biochemical parameters. For blood plasma assessment, tubes were centrifuged for 5 min at 3,000 rpm. Then, glass tubes were broken, and the resultant blood plasma was emptied into sterile micro tubes for further analysis. Serum biochemistry determinations included cholesterol, AST and ALT tests were made using an autoanalyser (Cobas Integra System, France). Hormonal Data tests include T3, Thyroxin, and TSH were made by Vidas Biomerieux system (Electroimmuno Analyzer, France) Strik et al., [2007]).

### Statistical analysis

The normality of the data was evaluated by applying the Kolmogorov–Smirnov test. For each normally distributed parameter analyzed the mean and standard deviation were calculated. In addition, Pearson’s coefficient was used for linear correlation (r) between variables at P < 0.05.

## Result

In order to establish reference ranges, the fish samples were subjected to an external examination and healthy fish were used. The average weight and total length of fish sample were 25.35 ± 5.30 g and 129.24 ± 9.76 mm, respectively (Table 1). Water temperature was 28°C, pH was 8.3, dissolved O_2_ was 6.8 mg L^−1^, total ammonia nitrogen (TAN) was < 0.5 mg L^−1^.

**Table 1:** **Biological and biochemical parameters (Mean ± SD) and range recorded for** ***L. klunzingeri*** **(n = 87)**

***Parameters***	***N***	***Mean ± S.D***	***Minimum***	***Maximum***
Weigh(g)	87	25.35 ± 5.30	13.27	48.77
Total length(cm)	87	129.24 ± 9.76	105.00	151.00
Forked length(cm)	87	119.13 ± 9.18	95.00	140.00
TSH(nmol/L)	87	0.03 ± 0.01	0.02	0.06
Cholesterol(mg/di)	87	177.28 ± 40.75	115.00	241.00
Thyroxin(ng/ml)	87	76.58 ± 28.26	41.00	124.00
T3(ng/ml)	87	0.96 ± 0.58	0.4	1.87
ALT(U/L)	87	1.71 ± 0.68	1.00	3.00
AST(U/L)	87	49.81 ± 5.25	41.00	59.00

The mean, minimum and maximum plasma parameters of *L. klunzingeri* are given in Table 1. There were correlations among studied factors in serum, A significant positive correlation (*P* < 0.01) was found between AST and Cholesterol. ALT had a significant and positive relationship with Cholesterol and AST (*P* < 0.01). Thyroxin also had a significant and positive relationship with cholesterol (*P* < 0.01) and AST (*P* < 0.01). The results revealed reveres correlation between Thyroxin with TSH (*P* < 0.01). T3 also had a significant and reveres correlation with TSH (*P* < 0.01) (Table 2).

**Table 2:** **Correlation between biological and biochemical parameters in** ***L. klunzingeri***

***Parameters***	***Total length***	***Forked length***	***Weight***	***Cholesterol***	***AST***	***ALTt***	***TSH***	***Thyroxin***
Forked length(cm)	.971^**^							
Weight(g)	.829^**^	.835^**^						
Cholesterol(mg/di)	.023	.027	.004					
AST(U/L)	.004	.024	-.003	.628^**^				
ALT(U/L)	-.019	-.012	-.002	.593^**^	.883^**^			
TSH(nmol/L)	-.026	-.044	-.007	-.803^**^	-.415^**^	-.399^**^		
Thyroxin(ng/ml)	-.005	.000	-.018	.972^**^	.644^**^	.582^**^	-.791^**^	
T3(ng/ml)	-.005	-.003	-.031	.981^**^	.700^**^	.639^**^	-.779^**^	.984^**^

**. Correlation is significant at the 0.01 level.

## Discussion

The study of blood parameters is one of the most valuable diagnostic tools because it has been shown that the physiological values of these parameters are species-specific and age-dependent ([Anver 2004], Darvish Bastami et al. [2009]). One of the difficulties in assessing the state of health of natural fish populations has been the paucity of reliable reference ranges of the normal condition. In pursuant to this goal, many fish physiologists have turned to studies of hematology, probably because this area has proved to be a valuable diagnostic tool in evaluating health. Although fish hematology continues to offer the potential of a valuable tool, progress in establishing normal range values for blood parameters has been slow, and literature in this area is isolated and often incomplete. The biochemical profile can also provide important information on the condition of the organism (Anver, [2004]). Accordingly, haematology and serum biochemistry data are of immense importance to help managers monitor the health of both captive and wild population of these species, especially in aquaculture, water quality assessment and etc. The use of a biochemical approach has been advocated to provide an early warning of potentially damaging changes in stressed fish. In toxicological studies of acute exposure, changes in concentrations and enzyme activities often directly reflect cell damage in specific organs (Casillas et al. [1983]). Blood parameters included plasma enzyme activities (aspartate aminotransferase (AST), alanine aminotransferase (ALT), alkaline phosphatase (ALP), glucose (GLU) and total protein (TP)) of Mullets (*Liza saliens*) were determined by C. Fernandez et al. ([2008]) in Esmoriz–Paramos lagoon, Portugal. Authors expressed that the higher glucose and protein contents observed in *Liza saliens* caught in the lagoon were consistent with a stress response and the measurement of plasma AST activity could be a sensitive indicator of lagoon fish stress.

Thyroid hormones are produced upon activation of the neuroendocrine hypothalamo-pituitary-thyroid (HPT) axis (Blanton and Specker, [2007]; Zoeller et al., [2007]). Under hypothalamic control, the pituitary secretes thyroid-stimulating hormone (TSH) which proceeds to the thyroid gland to activate synthesis of thyroxine (T4) and triiodothyronine (T3). T4 generally represents >95% of the thyroid hormone output and it is typically present in higher quantities than T3 in the blood circulation, with the higher T4 concentrations serving as a pool of prohormone that can be converted into T3 in target tissues (Eales, [2006]; Zoeller et al., [2007]). Our results also show that T4 and T3 were 76.58 ± 28.26 and 0.96 ± 0.58, respectively. Thyroid hormones are essential for early development in fishes, including larval–juvenile transitions and induction of metamorphosis (Blanton and Specker, [2007]; Klaren et al., [2008]). Thyroid hormones are also deposited into the yolk of fish eggs, and used during subsequent embryonic development (Leatherland, [1989]). Disruption of thyroid function can have severe consequences as thyroid hormones play an important role in the maintenance of a normal physiological status in vertebrates. In adult fish, thyroid hormones are of primary importance in the regulation of such fundamental physiological processes as growth, nutrient utilization, and reproduction. Fish grow faster and are healthier when thyroid hormone levels are adequate (Power et al., [2001]; Yamano, [2005]).

AST and ALT belong to the plasma non-functional enzymes which are normally localized within the cells of liver, heart, gills, kidneys, muscle and other organs. It is also considered to be important in assessing the state of the liver and some other organs. Their presence in blood plasma may give information on tissue injury or organ dysfunction. Monitoring of liver enzymes leakage into the blood has proved to be a very useful tool in liver toxic studies (Salah El-Deen & Rogeps, [1993]). In *L. klunzingeri* average of AST and ALT were: 49.81 ± 5.25 and 1.71 ± 0.68, respectively.

Cholesterol is major degradation products and indicators of carbohydrate, lipid and protein metabolism (Kaplan et al. [1988]). In *L. klunzingeri* minimum and maximum of cholesterol was: 115–241. Blood parameters among fish species may be affected by sampling technique, analyses methods, age, habitat, and diet (Sakamoto et al. [2001]). Therefore, values reported here will be useful for the early detection, identification, and monitoring of diseases and sub lethal conditions in this species.

## Authors’ information

MM is a scientific member of Environment Department of Islamic Azad University, Bandar Abbas Branch, MA and ME are Master student of Fisheries Department, KA is scientific member of Iranian National Institute for Oceanography and RE is Pathology lab technician.
